# Higher expression of TNFα-induced genes in the synovium of patients with early rheumatoid arthritis correlates with disease activity, and predicts absence of response to first line therapy

**DOI:** 10.1186/s13075-016-0919-z

**Published:** 2016-01-20

**Authors:** Aurélie De Groof, Julie Ducreux, Frances Humby, Adrien Nzeusseu Toukap, Valérie Badot, Costantino Pitzalis, Frédéric A. Houssiau, Patrick Durez, Bernard R. Lauwerys

**Affiliations:** Pôle de Pathologies Rhumatismales Inflammatoires et Systémiques, Institut de Recherche Expérimentale et Clinique, Université catholique de Louvain, Avenue Hippocrate 10, B2.5390, 1200 Brussels, Belgium; Centre for Experimental Medicine and Rheumatology, John Vane Science Centre, William Harvey Research Institute, St. Bartholomew’s and Royal London School of Medicine London, London, UK; Department of Rheumatology, Cliniques Universitaires Saint-Luc, Brussels, Belgium; Service de Rhumatologie, Hôpital Erasme, Brussels, Belgium

**Keywords:** Rheumatoid arthritis, Early rheumatoid arthritis, Synovitis, TNF-alpha

## Abstract

**Background:**

IL6-related T cell activation and TNFα-dependent cell proliferation are major targets of therapy in the RA synovium. We investigated whether expression of these pathways in RA synovial biopsies is associated with disease activity and response to therapy.

**Method:**

Correlation and gene set enrichment studies were performed using gene expression profiles from RA synovial biopsies. Immunostaining experiments of GADD45B and PDE4D were performed on independent additional sets of early untreated RA samples, obtained in two different centers by needle-arthroscopy or US-guided biopsies.

**Results:**

In 65 RA synovial biopsies, transcripts correlating with disease activity were strongly enriched in TNFα-induced genes. Out of the individual variables used in disease-activity scores, tender joint count, swollen joint count and physician’s global assessment, but not CRP or patient’s global assessment displayed a similar correlation with the expression of TNFα-dependent genes. In addition, TNFα-induced genes were also significantly enriched in transcripts over-expressed in synovial biopsy samples obtained from poor-responders to methotrexate or tocilizumab, prior to initiation of therapy.

GADD45B (induced by TNFα in monocytes) and PDE4D (induced by TNFα in FLS) immunostaining was significantly higher in overall poor-responders to therapy in 46 independent baseline samples obtained from early untreated RA patients prior to initiation of therapy. GADD45B (but not PDE4D) immunostaining was significantly higher in the sub-group of patients with poor-response to methotrexate therapy, and this was confirmed in another population of methotrexate-treated patients.

**Conclusion:**

Higher expression of TNFα-induced transcripts in early RA synovitis is associated with higher disease activity, and predicts poor response to first-line therapy. That over-expression of TNFα-induced genes predicts poor-response to therapy regardless of the drug administered, indicates that this molecular signature is associated with disease severity, rather than with specific pathways of escape to therapy.

**Electronic supplementary material:**

The online version of this article (doi:10.1186/s13075-016-0919-z) contains supplementary material, which is available to authorized users.

## Background

Access to synovial material from patients with rheumatoid arthritis (RA) provides unique opportunities to investigate local molecular and cellular mechanisms of inflammation, and their links with clinical outcomes. Thus, histological and gene expression studies on RA synovial biopsies recently showed the presence of distinct, although partly overlapping, phenotypes: myeloid, lymphoid, low inflammatory and fibroid, based on most prevalent cell type, and related gene expression patterns. Concentrations of serum markers associated with these phenotypes were found to be informative about response to therapy in a biomarker study performed in patients included in the ADACTA trial. Thus, a high baseline serum sICAM1/CXCL13 ratio, reflecting a myeloid phenotype, predicted a higher probability of response to TNF blockade (American College of Rheumatology 50 % response (ACR50) 42 % versus 13 %), while a high CXCL13/sICAM1 ratio (lymphoid phenotype) was rather associated with better response rates to tocilizumab therapy (ACR50 69 % versus 20 %) [1, 2].

Using high-density and customized low-density gene expression platforms, we also found heterogeneous expression of T cell (lymphoid)-associated transcripts in RA synovial tissue. In a group of 32 biopsies obtained in treated and untreated patients, we observed that expression of these transcripts displayed a significant, albeit moderate (0.40 < *r* <0.55) correlation with disease activity (disease activity score in 28 joints-C reactive protein (DAS28-CRP)) [3, 4]. In other studies, we evaluated the effects of therapies on global gene expression patterns in prospective synovial biopsy samples obtained prior to and 3 months after initiation of therapy with methotrexate, tocilizumab, rituximab or adalimumab. We showed that methotrexate, tocilizumab and rituximab display very similar molecular effects in RA synovitis, characterized by a decrease in T cell activation genes [5, 6]. By contrast, TNF blockade resulted in a decrease in the expression of transcripts involved in cell proliferation and inflammation. Interestingly, higher baseline expression of TNFα-induced transcripts in RA synovial tissue was associated with decreased responses to TNF blockade in methotrexate-resistant patients [7, 8]. These observations probably indicate that, in some cases, tissue impregnation in TNFα is too high to be blocked using standard TNF blockade regimens.

Overall, these observations indicate that expression of TNFα- or T cell-associated transcripts displays a large level of plasticity in RA synovitis, related to disease activity, and effects of therapy. We therefore undertook the present study, on existing sets of gene expression data generated in our laboratory, in order to investigate the impact of disease activity on synovial molecular pathways, and assess whether variations in synovial gene expression profiles are also informative about disease outcomes.

## Methods

### Gene expression data sets

Transcriptomic data (GeneChip Human Genome U133 Plus2.0.CEL files, Affymetrix) from 65 samples obtained by needle-arthroscopic knee synovial biopsy were used in the present analyses. These samples were obtained in untreated RA patients (<1 year disease duration for the majority of them), prior to and 3 months after initiation of tocilizumab (*n* = 13 and 12 samples, respectively) or methotrexate (*n* = 2 × 8 samples) therapy [GEO:GSE45867] (National Center for Biotechnology Information; http://www.ncbi.nlm.nih.gov/geo), and in RA patients resistant to TNF blockade, prior to and 3 months after administration of rituximab (*n* = 2 × 12 samples, GSE24742) therapy [5, 6]. All patients met the ACR/European League Against Rheumatism (EULAR) 2010 RA classification criteria, and the ACR 1987 revised classification for RA. Patients’ characteristics are displayed in Table 1.

**Table 1:** Patients’ characteristics (high-density transcriptomic studies)

Patients with early rheumatoid arthritis (*n* = 21)	
Baseline biopsies (number)	21
Follow-up (3 months) biopsies (number)	20
Age at baseline (mean ± SD, years)	51.4 ± 12.8
Gender (female/male, number)	16/5
Anti-citrullinated peptide antibodies status (% positive)	66.7 %
Rheumatoid factor status (% positive)	71.4 %
Disease duration (mean ± SD, years)	0.5 ± 1.1
Disease activity score in 28 joints-CRP in methotrexate group (*n* = 9)	
Baseline	4.51 ± 1.49
After 3 months	3.84 ± 1.61
After 6 months	3.10 ± 1.54
Good, moderate, poor responders (number at 3 months)	2, 3, 4
Good, moderate, poor responders (number at 6 months)	5, 2, 2
Disease activity score in 28 joints-CRP in tocilizumab group (*n* = 12)	
Baseline	4.50 ± 1.10
After 3 months	2.35 ± 0.95
After 6 months	2.17 ± 1.14
Good, moderate, poor responders (number at 3 months)	7, 4, 1
Good, moderate, poor responders (number at 6 months)	9, 0, 3
Rheumatoid arthritis patients refractory to TNF blockade	
Baseline biopsies, number	12
Follow-up (3 months) biopsies, number	12
Age at baseline (mean ± SD, years)	54.6 ± 16.1
Gender (female/male, number)	9/3
Anti-citrullinated peptide antibodies status (% positive)	50 %
Rheumatoid factor status (% positive)	41.7 %
Patients receiving concomitant methotrexate therapy	100 %
Disease activity score in 28 joints-CRP at baseline	5.80 ± 1.32
Disease activity score in 28 joints-CRP 3 months after administration of rituximab treatment	4.48 ± 1.28

*Disease activity score in 28 joints-CRP* disease activity score in 28 joints using the C-reactive protein level

### Student’s *t* tests, correlation and pathway analyses

Selected .CEL files were uploaded on GeneSpring software (Agilent Technologies), and fluorescence intensity data were normalized using robust multi-array analysis. Normalized, log2-transformed gene expression data were exported on Excel (Microsoft) in order to calculate Pearson *r* correlation coefficients with disease activity score in 28 joints using the C-reactive protein level (DAS28-CRP), simplified disease activity index (SDAI), clinical disease activity index (CDAI) or each individual component of these scores. Student’s *t* test (without correction for multiple comparisons) was performed on baseline gene expression data from good versus poor responders to therapy, using GeneSpring. A cutoff value of 1.5 was further used to discriminate genes over-expressed in poor versus good responders to therapy. Pathway analyses were performed with lists of probe sets displaying a Pearson *r* correlation coefficient >0.5 with DAS28-CRP, using the Database for Annotation, Visualization and Integrated Discovery (DAVID), an application that interrogates functional annotation databases (Gene Ontology, KEGG, Biocarta and InterPro), and finds overrepresented biologic themes within a group of genes (http://david.abcc.ncifcrf.gov) [9].

### Gene set enrichment analyses

Lists of selected probe sets were uploaded on GeneSpring, and their eigenvalues were calculated in several experiments. Eigenvalues are the percentages of variation in gene expression data that are explained by the principal component of the experiment (whatever the amplitude of the variation). The influence of IL6 was assessed using synovial biopsy samples prior to and after administration of tocilizumab, an anti-IL6R antibody, using GSE45867 data. The effects of TNFα were analyzed using online available gene expression data (GSE38351) obtained from monocytes (*n* = 3) cultured for 1.5 hours in the presence or the absence of TNFα [10], and gene expression data produced in our laboratory on synovial fibroblast-like synoviocytes (FLS) cultured overnight in the presence or the absence of TNFα (10 ng/ml). For this experiment, FLS were purified from synovial biopsies from one patient with osteoarthritis, and two patients with RA, as previously described [8]. After overnight incubation, cells were detached and total RNA was extracted using the Nucleospin RNA II extraction kit (Macherey-Nagel), including DNase treatment of the samples. Complementary RNA (cRNA) was synthetized, biotin-labeled and fragmented according to a standard Affymetrix procedure (Genechip 3′ IVT Express kit, Affymetrix), and GeneChip Human Genome U133 Plus2.0 arrays were hybridized overnight with 10 μg cRNA. The slides were washed and stained using a EukGE-WS2v5 fluidics protocol on a GeneChip fluidics Station 450, before being scanned on a GeneChip Scanner 3000 (Affymetrix). The .CEL files corresponding to this experiment are available [GEO:GSE15615 and GSE73764].

### Immunohistochemistry experiments

GADD45B and PDE4D immunolabeling experiments were performed using paraffin-embedded synovial sections obtained in two different cohorts of patients with early RA. Knee synovial biopsies were obtained by needle arthroscopy in a first population of 46 patients with early (disease duration <1 year) ACR/EULAR 2010 classification criteria-positive RA. These patients were untreated at the time of the biopsy (except for use of non steroidal anti-inflammatory drugs (NSAIDs)). Next, 17 of them received oral methotrexate (15–20 mg/week), while the other patients received biological original disease-modifying anti-rheumatic drugs (DMARDs), alone or in combination with oral methotrexate (see Additional file 1).

Ultrasound (US)-guided biopsies were obtained in 35 additional patients with early (disease duration <1 year) untreated ACR/EULAR 2010 classification criteria-positive RA, prior to initiation of methotrexate therapy (see Additional file 2). Response to therapy was determined in both cohorts using EULAR response criteria, based on DAS28-CRP evaluations at baseline, and 6 months after initiation of therapy.

Knee synovial biopsies were also obtained by needle arthroscopy from the knee of untreated patients with psoriatic arthritis (*n* = 9), and osteoarthritis (*n* = 12). After removal of paraffin, sections were incubated in a sodium citrate buffer (ImmunoDNA Retriever with citrate, BioSB) and heated in a bain-marie for 60 minutes at 98 °C to retrieve the antigenic sites. Endogenous peroxidase was inactivated with Peroxydase Blocking Reagent (Dako), and nonspecific binding was blocked by 15-minute incubation with 1 × Envision buffer (Dako) containing 1 % normal human IgG, 5 % bovine serum albumin and 2 % milk. Sections were then incubated overnight at 4 °C with primary antibody. The following antibodies were used: GADD45B (rabbit polyclonal, 1/100 dilution, Sigma) and PDE4D (rabbit polyclonal, 1/2,000 dilution, Sigma). After three washes in 1 × Envision buffer, specifically bound antibodies were labeled with anti-rabbit Envision-HRP labeled polymer for 1 hour at room temperature, and peroxidase activity was revealed by 10-minute incubation with diaminobenzidine (Dako). As a final step, sections were washed in tap water and lightly counterstained with hematoxylin.

Slides were scanned on a Mirax station (Zeiss) and quantitative analysis of the immunostained sections was performed using Frida software [11], in accordance with the digital image analysis process [12]. Six digitized pictures (magnification × 400) were obtained for each slide by one operator who was blinded to the identity of the specimens. Each picture included a lining and sublining region when possible. When the distribution of the staining was heterogeneous, the pictures were taken in order to be representative of the whole slide. The surface staining (S) and the surface of the nuclei (N) were determined for each image, and the area of staining then was normalized by calculating the ratio of S to N, and averaging it for each slide. When the quality of the tissue was insufficient to obtain enough material for quantification, the sample was discarded. For GADD45B, this was the case in 6 out of 46 knee and 9 out of 35 US-guided biopsies. For PDE4D, this was the case in 3 out of 46 knee and 7 out of 35 US-guided biopsies. Between-group differences were assessed using the Kruskal–Wallis and Mann–Whitney tests.

The study was approved by the Ethical Committee of the Université catholique de Louvain, and informed consent was obtained from all patients.

## Results

**Fig. 1:**
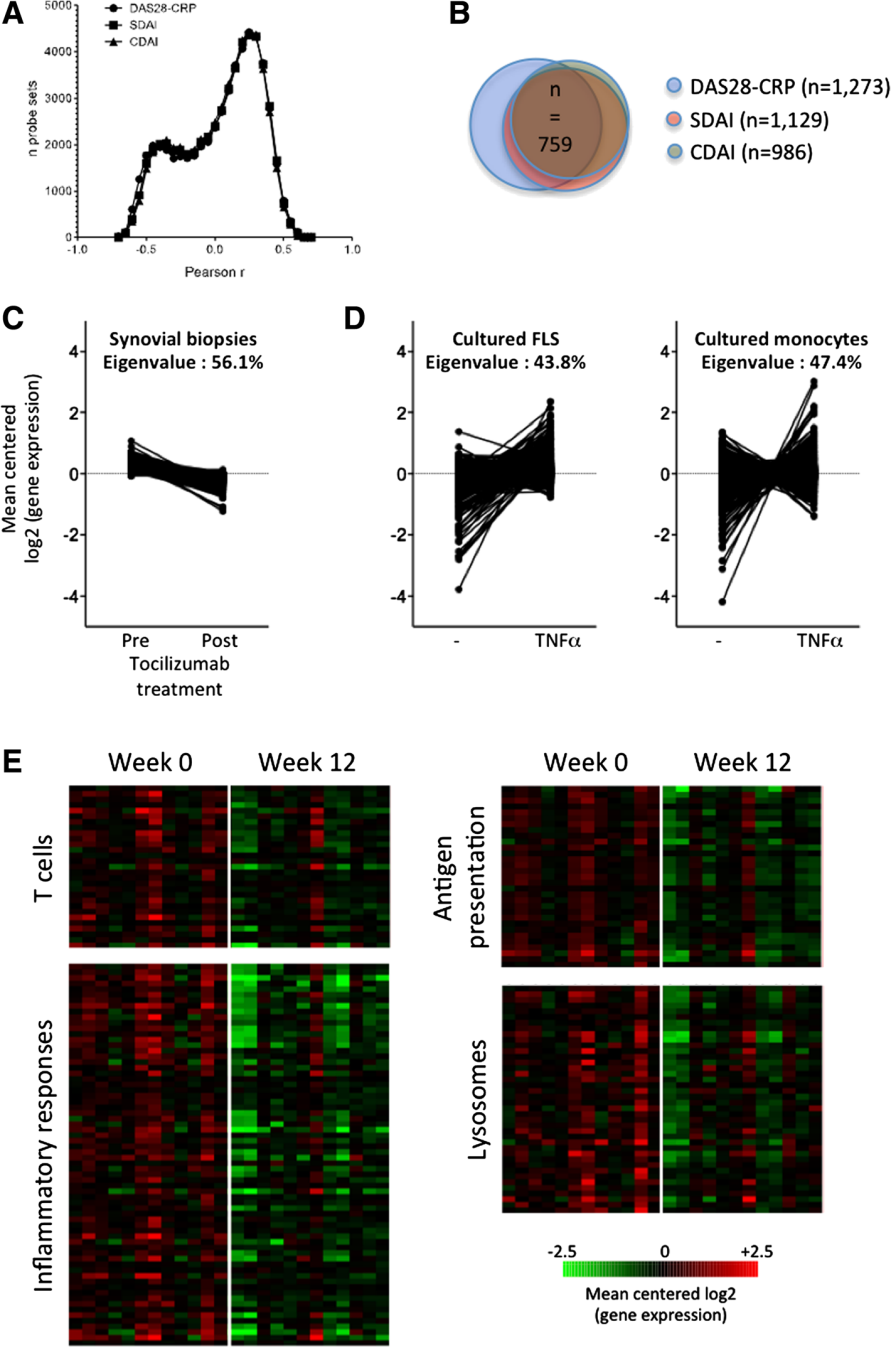
Correlations between clinical disease activity indices and the expression of 51,452 probe sets in 65 synovial biopsy samples from untreated and treated patients with rheumatoid arthritis (RA). **a** Distribution of Pearson *r* correlation coefficients between disease activity score in 28 joints assessed by C-reactive protein level (*DAS28*-CRP)/simplified disease activity index (*SDAI*)/clinical disease activity index (*CDAI*) and synovial gene expression levels. **b** Overlap between probe sets displaying a Pearson *r* correlation coefficient >0.5 with DAS28-CRP, SDAI and CDAI. **c**, **d** Gene set enrichment analyses and eigenvalues calculations using probe sets displaying a Pearson *r* correlation coefficient >0.5 with DAS28-CRP indicate that the majority of them are downregulated by tocilizumab in synovial tissue in RA (**c**), and a large proportion are induced by TNFα in cultured fibroblast-like synoviocytes (*FLS*) or monocytes (**d**). **e** Pathway analyses (http://david.abcc.ncifcrf.gov) indicate that transcripts displaying a Pearson *r* correlation coefficient >0.5 with DAS28-CRP are significantly enriched in genes associated with T cell activation, inflammatory responses, antigen presentation and lysosomes. Probe sets belonging to these pathways are downregulated by tocilizumab in RA synovial tissue

In order to identify molecular pathways that drive disease activity in RA synovitis, we calculated the correlation coefficients of clinical disease activity scores (DAS28-CRP, CDAI, SDAI) with the expression levels of 51,452 individual probe sets in 65 synovial biopsies from 33 different RA patients (collected prospectively before and after administration of several therapies; see Table 1). Around 1,000 transcripts displayed a correlation coefficient (Pearson *r*) ≥0.5 with disease activity scores (Fig. 1a). All three disease activity indices generated strongly overlapping results (Fig. 1b). Gene set enrichment analyses indicated that almost all these transcripts are downregulated by tocilizumab (anti-IL6R antibody) in the synovium in early RA, which indicates that they are, directly or indirectly, IL6-dependent (Fig. 1c). A large proportion of these transcripts is also TNFα-dependent, as they are induced by the addition of TNFα in peripheral blood monocyte and/or FLS cell cultures (Fig. 1d). Additional pathway analyses indicated that these transcripts are significantly enriched in T cell activation, antigen presentation, inflammation and lysosome genes, many of which are known tocilizumab targets in the synovium in RA (Fig. 1e).

**Fig. 2:**
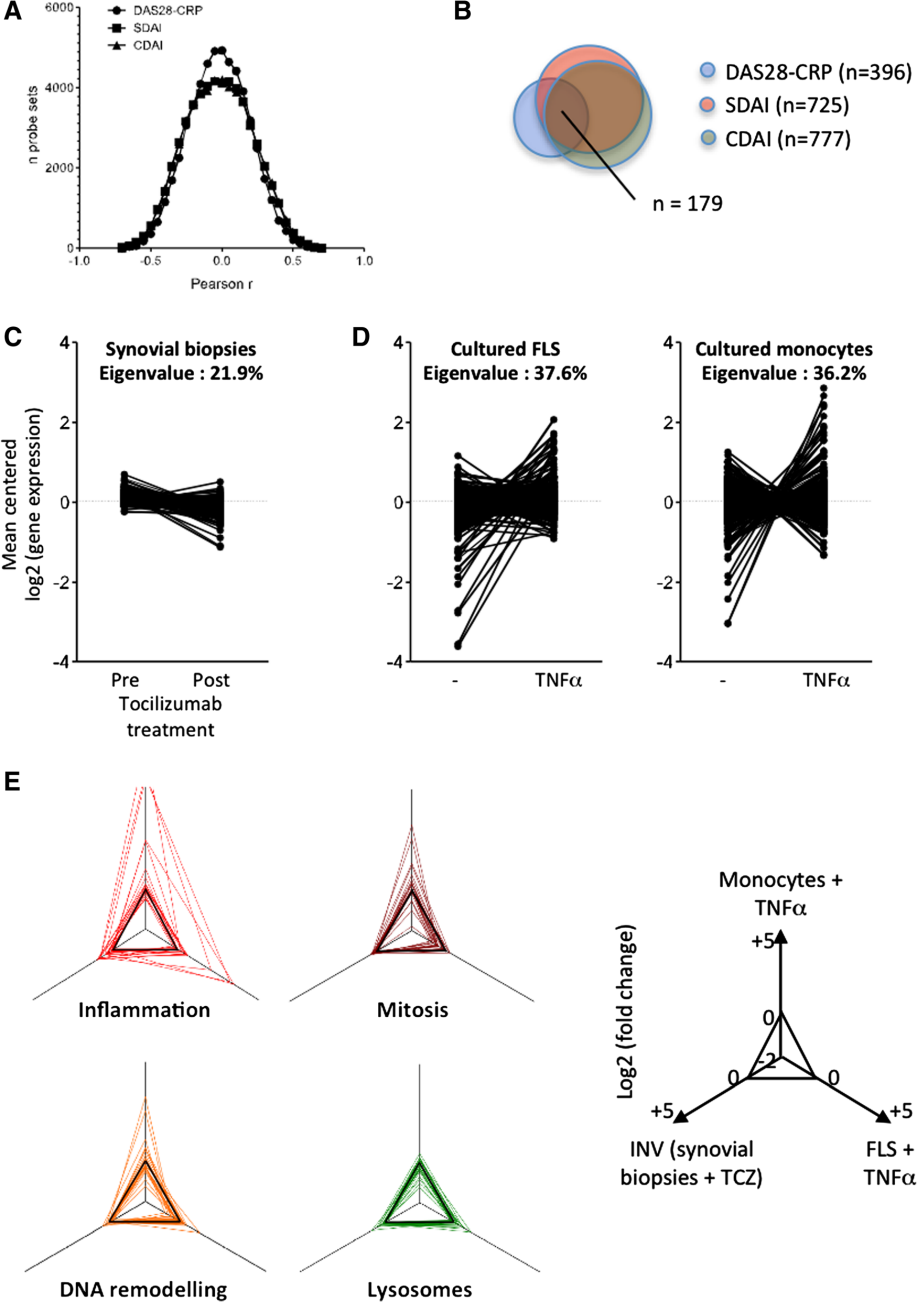
Correlations between clinical disease activity indices and the expression of 51,452 probe sets in 21 synovial biopsy samples from untreated patients with early rheumatoid arthritis (RA). **a** Distribution of Pearson *r* correlation coefficients between disease activity score in 28 joints assessed by C-reactive protein level (*DAS28*-CRP)/simplified disease activity index (*SDAI*)/clinical disease activity index (*CDAI*) and synovial gene expression levels. **b** Overlap between probe sets displaying a Pearson *r* correlation coefficient >0.5 with DAS28-CRP, SDAI and CDAI. **c**, **d** Gene set enrichment analyses and eigenvalues calculations using probe sets displaying a Pearson *r* correlation coefficient >0.5 with DAS28-CRP indicate that a subset of these transcripts is downregulated by tocilizumab in synovial tissue in RA (**c**), and a large proportion is induced by TNFα in cultured fibroblast-like synoviocytes (*FLS*) or monocytes (**d**). **e** Pathway analyses (http://david.abcc.ncifcrf.gov) indicate that transcripts displaying a Pearson *r* correlation coefficient >0.5 with DAS28-CRP are significantly enriched in genes associated with inflammation, mitosis, DNA remodeling and lysosomes. The radar plots indicate how the transcripts belonging to these pathways are influenced by tocilizumab in synovial biopsies, and by TNFα in cultured monocytes and FLS. *INV* inverse (a negative effect of tocilizumab appears as positive and suggests a positive effect of IL6)

Samples used in this first study were obtained from untreated patients, but also from the same patients 3 months after initiation of methotrexate, tocilizumab or rituximab therapy. In order to avoid biases associated with the use of treated samples, we repeated our analyses, this time only in samples (*n* = 21) obtained from untreated patients. Again, all three clinical indices of disease activity generated strongly overlapping results (Fig. 2a and b). When looking at transcripts correlating with individual components of the disease activity scores (tender joint count, swollen joint count, patient’s global assessment, physician’s global assessment, CRP), we found that transcripts correlating with tender joint count, physician’s global assessment and swollen joint count displayed the strongest overlap with the global scores (see Additional file 3). Gene set enrichment analyses were performed using the transcripts displaying *r* ≥0.5 with DAS28-CRP. The changes in eigenvalues (i.e., the percentage of variation in gene expression explained by the principal component of the experiment), and the changes in the amplitude of the effects compared to the initial analyses showed that these transcripts are much less IL6-dependent, but still comparably TNFα-dependent (Fig. 2c and d). Pathway analyses indicated a significant enrichment in the following functional terms: inflammation, mitosis, DNA remodeling and lysosomes. Transcripts belonging to some of these pathways (mitosis, DNA remodeling) are strongly TNFα-dependent, while transcripts belonging to the inflammation pathway are both TNFα-dependent and IL6-dependent (Fig. 2e).

**Fig. 3:**
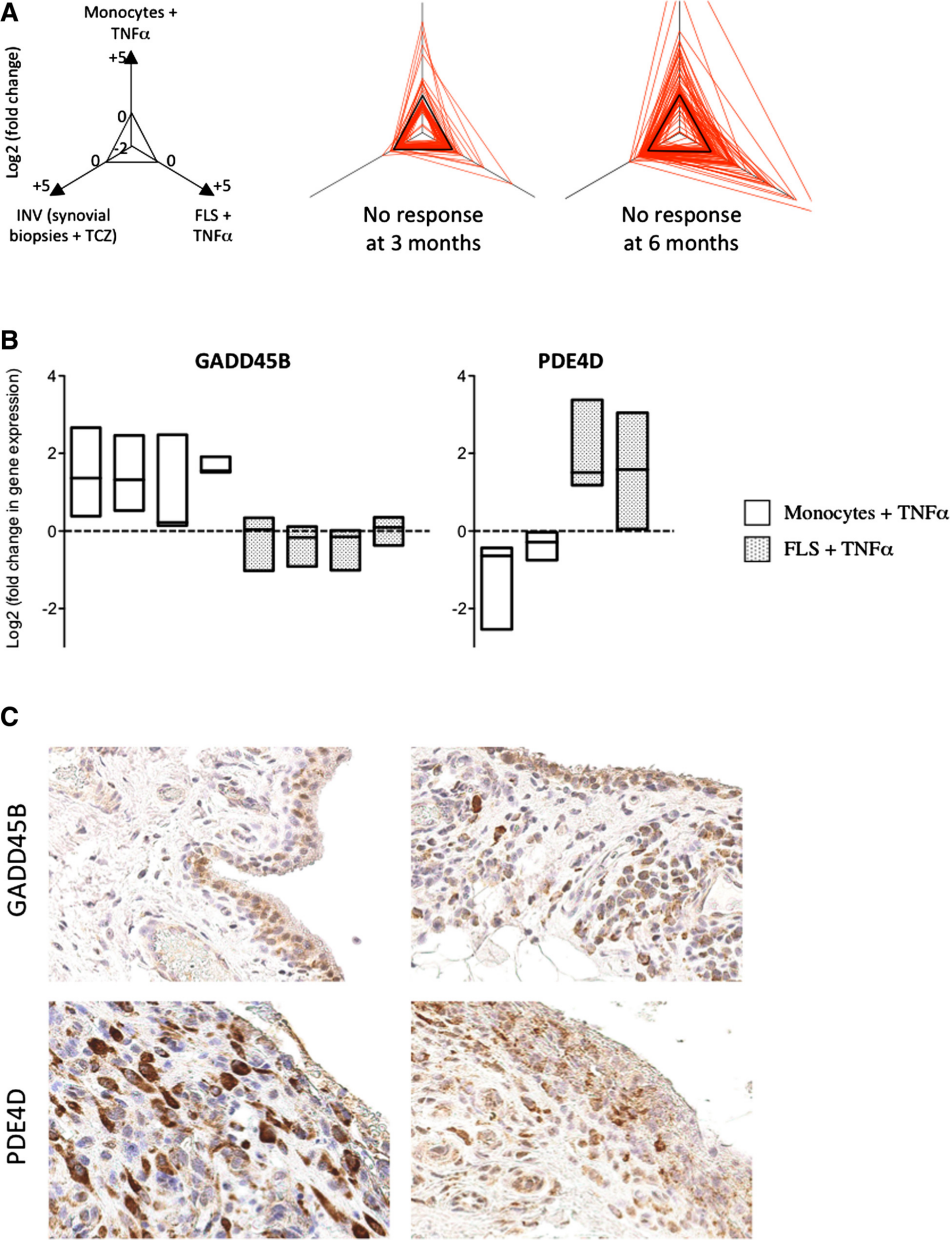
Higher synovial expression of TNFα-induced transcripts is associated with overall absence of response to therapy in early rheumatoid arthritis (RA). **a** Baseline synovial gene expression profiles were compared in poor versus good responders to either tocilizumab or methotrexate therapy at 3 months (4 non-responders versus 9 good responders), and at 6 months (4 non-responders versus 15 good responders). The radar plots indicate how transcripts over-expressed in poor responders (*p* <0.05, >1.5-fold change in gene expression) are influenced by tocilizumab in synovial biopsies, and by TNFα in cultured monocytes and fibroblast-like synoviocytes (*FLS*). *INV* inverse (a negative effect of tocilizumab appears as positive and suggests a positive effect of IL6). **b** Fold changes in the expression of GADD45B and PDE4D encoding probe sets obtained from HGU133 Plus2.0 transcriptomic data generated in TNFα-stimulated versus non-stimulated monocytes (*n* = 3) and FLS (*n* = 3). *Box plots* represent minimum, maximum and median log2 (fold changes) compared to non-stimulated cells. **c** Typical GADD45B and PDE4D immunostaining pictures (original magnification × 400) obtained in early RA synovial biopsies, illustrative of marked inter-individual variations in protein expression

**Fig. 4:**
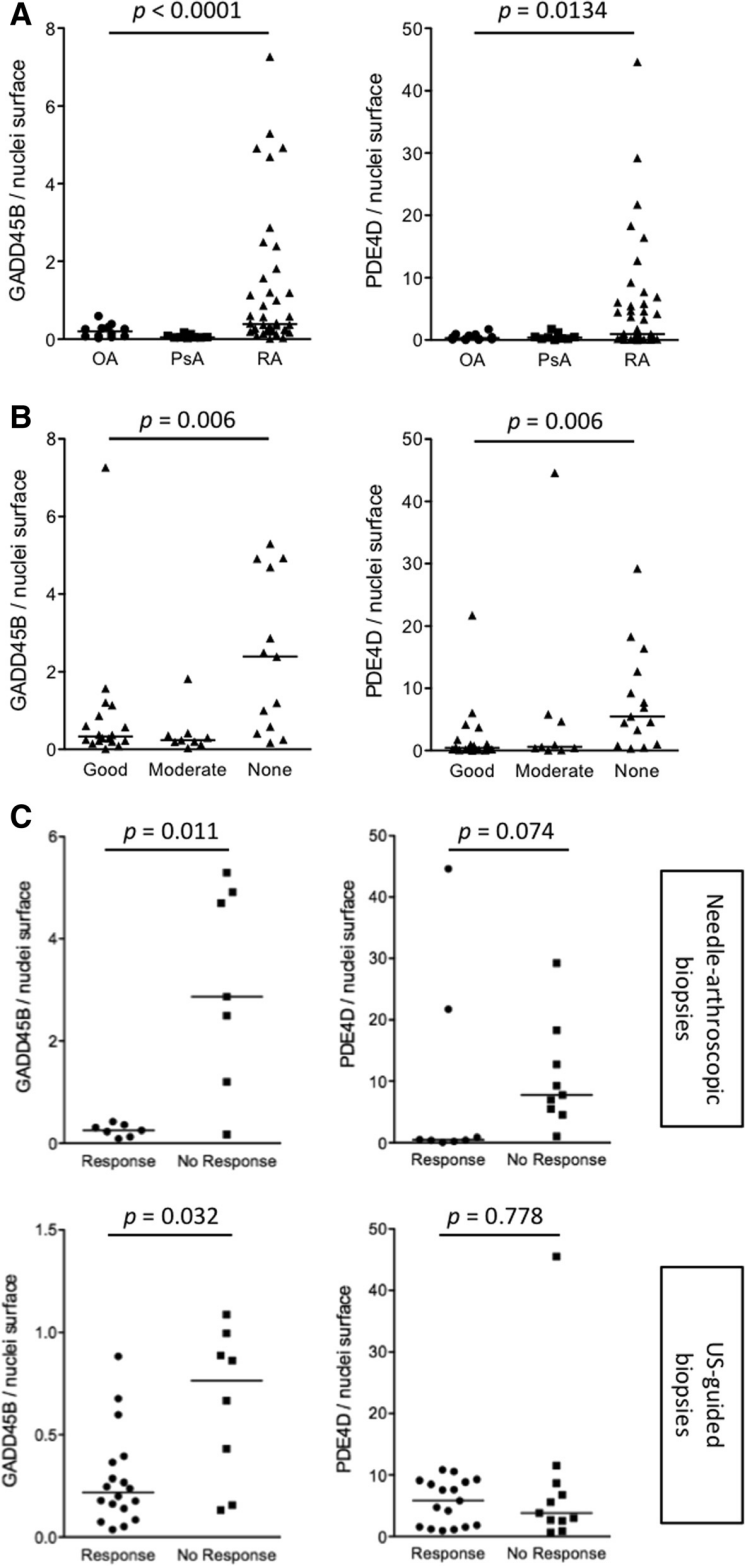
Quantification of GADD45B and PDE4D immunostaining in baseline synovial biopsies from patients with early rheumatoid arthritis (RA), osteoarthritis and psoriatic arthritis. **a** Needle-arthroscopic synovial biopsies from untreated patients with early RA (*n* = 46), psoriatic arthritis (*n* = 9) and osteoarthritis (*n* = 12) were stained with polyclonal antibodies directed at GADD45B and PDE4D. Data are represented as the ratio of GADD45B or PDE4D surface staining to staining of the nuclei. *Horizontal bars* represent the median values. *P* values were calculated using Kruskal–Wallis test. **b** GADD45B and PDE4D immunostaining in needle-arthroscopic synovial biopsies obtained in patients with early RA prior to initiation of therapy. Patients are distributed as good, moderate and poor responders to therapy according to European League Against Rheumatism (EULAR) response criteria at 6 months. *P* values were calculated using Kruskal–Wallis test. **c** GADD45B and PDE4D immunostaining in needle-arthroscopic (sub-group of population displayed in **a** and **b**) and ultrasound-guided biopsies obtained in patients with early RA prior to initiation of methotrexate therapy. Patients are distributed as responders or non-responders according to EULAR response criteria at 6 months. *P* values were calculated using Mann–Whitney test

Next, we wondered whether over-expression of TNFα-induced genes in baseline synovial tissue was also associated with overall disease evolution. Six months after the biopsy, 4 out of 21 patients were non-responders and 15 were good responders to either methotrexate or tocilizumab therapy. As shown in Fig. 3a, transcripts over-expressed in the synovium of non-responders (see Additional file 4) were significantly enriched in TNFα-induced genes. In order to confirm this observation, we performed immunohistochemistry experiments in two independent cohorts of synovial biopsies, using two TNFα-dependent markers: PDE4D, which is preferentially induced in TNFα-stimulated fibroblasts, and GADD45B, preferentially induced in TNFα-stimulated monocytes (Fig. 3b). We found that both molecules are strongly over-expressed in RA compared to osteoarthritis or psoriatic arthritis synovial biopsies (Fig. 4a). In addition, both GADD45B and PDE4D are significantly over-expressed in baseline synovial tissue of patients with early RA, who will not respond to methotrexate or original biological DMARDs given as first line monotherapy or combination therapy. Out of the 46 RA synovial biopsy samples used in these immunostaining experiments, 17 were taken prior to initiation of methotrexate monotherapy. In this subgroup of samples, baseline over-expression of GADD45B (but not PDE4D) was significantly associated with poor response to therapy. Similarly, baseline over-expression of GADD45B (but not PDE4D) was associated with poor response to methotrexate therapy in an independent set of US-guided synovial biopsies obtained in patients with early RA prior to initiation of therapy.

## Discussion

We studied correlations between clinical indices of disease activity and global gene expression levels in RA synovitis. We found that synovial transcripts correlating with DAS28-CRP are significantly enriched in TNFα-induced genes in early RA. In addition, high synovial expression of TNFα-induced transcripts is associated with poor response to therapy in early disease. Immunohistochemistry experiments on two independent cohorts of patients confirmed that baseline over-expression of GADD45B, a molecule induced by TNFα in monocytes in vitro, is associated with poor response to therapy in early RA.

Identification of molecular pathways driving disease activity in RA synovitis is an important step toward a better understanding of the complex pathogenesis of the disease. Hence, a careful approach to the data is warranted, in particular when the analytical process is based on correlative studies. In this perspective, our data illustrate nicely how methodological choices can influence the shape of such correlative results. Thus, our initial analyses, performed on 65 samples obtained before and after initiation of methotrexate, tocilizumab or rituximab therapy, point at a predominant role of IL6-derived mechanisms in driving disease activity in RA, as almost all the genes correlating (*r* ≥0.5) with DAS28-CRP are downregulated by tocilizumab therapy in RA biopsy samples. Yet, we recently demonstrated that all three drugs display very similar molecular effects in RA synovitis, characterized by a downregulation of T cell activation-associated genes, and a large panel of chemokine and cytokine transcripts [6]. Thus, not only tocilizumab, but also rituximab and methotrexate decrease the expression of IL6-dependent transcripts in RA synovitis. Because these three drugs also downregulate disease activity, inclusion of post-treatment samples in our initial analyses increased the odds of finding an association between IL6-dependent transcripts and disease activity, and this is what we observed.

We therefore repeated our analyses, excluding samples from treated patients. In synovial biopsies from patients with early (untreated) RA, transcripts correlating with DAS28-CRP (*r* ≥0.5) are significantly enriched in TNFα-dependent genes. Some of these genes are also induced by IL6, but to a lesser extent. The fact that disease activity score-associated transcripts are more enriched in TNFα-induced genes in early RA does not mean that TNFα plays a greater role in disease pathogenesis at this stage of the disease. Instead, it means that the variables with the highest weights in the disease activity scores in patients with active RA (e.g., number of tender and swollen joints) are more susceptible to direct influence of TNFα-dependent mechanisms. This could be due to the fact that TNFα-related mechanisms drive disease extension, resulting in higher numbers of affected joints. Alternatively, over-expression of TNFα-induced genes could make affected joints more susceptible to detection by the clinician. In this perspective, performing similar analyses with ultrasound rather than clinical scores would be of interest, in order to evaluate whether expression of TNFα-dependent transcripts in synovial biopsies correlate with higher gray-scale and power Doppler signals, hence a higher probability of being detected clinically.

One striking observation is that all three disease activity indices - DAS28-CRP, SDAI and CDAI - generated highly concordant results in the correlation analyses with synovial gene expression data. This is not a surprise considering that these three indices are calculated based on an overlapping set of variables (tender joint count, swollen joint count, CRP, patient’s global assessment, physician’s global assessment). Comparing the results of the correlation analyses using the global indices, versus each of their individual components, it appears that the highest overlap with the global indices is found for the tender joint count (the variable with the highest weight in all the scores). The lowest overlap is found for patient’s global assessment, and CRP. Interestingly, there was also a large overlap found between the three clinical indices and physician’s global assessment, although this variable is not included in the DAS calculation, and has little weight in the SDAI and CDAI. Although one should not underestimate the risk of circularity in the reasoning, association of independent clinical indices of disease activity with synovial molecular signatures known to be involved in the pathogenesis of RA, provides strong extra-clinical support to the view that these scores adequately and equally capture information that is truly representative of the disease.

From a clinical point of view, assessment of disease activity using synovial transcriptomic data is not the most valuable information one expects from synovial biopsies, considering how readily disease activity is evaluated at the bedside by a trained clinician. Instead, evaluation of variables associated with less favorable disease outcomes, and a decreased probability of response to therapy, is more helpful, not only in daily clinical practice, but also in the context of patient stratification strategies in clinical trials. In our previous work we demonstrated that baseline over-expression of TNFα-induced genes in the synovium from patients with DMARD-resistant RA was associated with poor response to TNF blockade [7, 8]. Our present gene expression and immunohistochemistry results obtained from different cohorts of patients indicate that baseline synovial over-expression of TNFα-induced genes is also associated with poor response to therapy, in particular methotrexate, in early RA. The fact that greater synovial expression of TNFα-dependent molecules is associated with poor therapeutic outcomes regardless of the drug received by the patient, probably indicates that this molecular signature is associated with overall disease severity, rather than with activation of specific pathways of escape to therapies. Translating this hypothesis in clinical terms would lead to the speculation that patients with high synovial expression of TNFα-induced genes do not necessarily need alternative therapies, but stronger therapies, a statement that obviously needs to be verified in further studies (also bearing in mind that estimation of not only drug efficacy, but also toxicity, needs to be incorporated into personalized treatment algorithms).

In our immunohistochemistry confirmation studies, we selected GADD45B and PDE4D, because of their in vitro specific pattern of induction in monocytes versus FLS, respectively. Both molecules were already subject to scrutiny in the context of RA. Thus, GADD45B is mainly expressed in synovial CD68 positive macrophages in RA, in response to pro-inflammatory cytokines, such as TNFα [13, 14]. PDE4 isoforms regulate intracellular cAMP concentrations, and knowledge about their involvement in arthritis mainly originates from clinical studies on apremilast, a specific PDE4 inhibitor. Apremilast is registered for the treatment of psoriasis and psoriatic arthritis [15]; however, the drug does not have superior efficacy compared to placebo in methotrexate-resistant RA patients [16]. In our results, baseline over-expression of both GADD45B and PDE4D is associated with overall poor response to therapy in a group of untreated patients with early RA. When we restricted the analyses to methotrexate therapy, the association remained significant for baseline GADD45B over-expression in two independent cohorts of patients with early RA.

## Conclusions

At the present time, stratification of RA patients in clinical trials is based on parameters (autoantibodies, radiographic evidence of erosions, acute phase reactants) that are associated with disease severity, yet are poorly discriminant on an individual basis. Our data provide a strong rationale for including synovial markers of disease severity in the stratification panels of clinical trials, in view of their ability to identify patients with a low overall probability of response to therapy. The development of minimally invasive techniques (needle arthroscopy and US-guided biopsy) potentially gives access to synovial material from any joint in ambulatory patients [17]. In addition, a large international effort is ongoing to standardize pre-analytical and analytical procedures for synovial biopsy sampling [18]. In this context, our results open perspectives in terms of a new molecular classification of patients with rheumatoid arthritis, resulting in a more precise evaluation of disease outcomes and the need for more aggressive therapies.

## Additional files

Additional file 1:
**Patients’ characteristics (immunohistochemistry studies on needle arthroscopic biopsies).** Characteristics of the patients with early rheumatoid arthritis (RA) included in the immunohistochemistry studies on needle arthroscopic biopsies. (DOCX 56 kb) Additional file 2:
**Patients’ characteristics (immunohistochemistry studies on ultrasound (US)-guided biopsies).** Characteristics of the patients with early rheumatoid arthritis (RA) included in the immunohistochemistry studies on US-guided biopsies. (DOCX 47 kb) Additional file 3:
**Numbers of probe sets displaying a positive correlation with global disease activity indices and each of their individual variables.** Overlap between probe sets displaying a Pearson *r* correlation coefficient >0.5 between tender joint count, swollen joint count, patient’s global assessment, physician’s global assessment, C-reactive protein (CRP) and global disease activity scores. (XLSX 37 kb) Additional file 4:
**Genes** **over-expressed** **in poor responders to therapy at baseline.** Lists of probe sets differentially expressed at baseline in synovial biopsies from patients with early rheumatoid arthritis (RA) in good versus poor responders to first line therapy at 3 and 6 months. (XLSX 293 kb)

## Abbreviations

ACR50: American College of Rheumatology 50 % response
ACR/EULAR: American College of Rheumatology/European League Against Rheumatism
ADACTA: Adalimumab Actemra trial
CDAI: clinical disease activity index
CRP: C-reactive protein
CXCL13: chemokine (C-X-C motif) ligand 13
DAS28-CRP: disease activity score in 28 joints using C-reactive protein level
DMARD: disease-modifying anti-rheumatic drug
DNA: Deoxyribonucleic acid
FLS: fibroblast-like synovial cells
GADD45B: growth arrest and DNA-damage-inducible, beta
GEO: Gene Expression Omnibus
HRP: horseradish peroxidase
IL6: interleukin 6
IL6R: interleukin 6 receptor
NSAID: non steroidal anti-inflammatory drug
PDE4D: phosphodiesterase 4D, cAMP-specific
RA: rheumatoid arthritis
RNA: Ribonucleic acid
SDAI: simplified disease activity index
sICAM1: soluble intercellular adhesion molecule 1
TNFα: tumor necrosis factor alpha
US: ultrasound

## References

[1] Pitzalis C, Kelly S, Humby F (2013). New learnings on the pathophysiology of RA from synovial biopsies. Curr Opin Rheumatol.

[2] Dennis G, Holweg CT, Kummerfeld SK, Choy DF, Setiadi AF, Hackney JA (2014). Synovial phenotypes in rheumatoid arthritis correlate with response to biologic therapeutics. Arthritis Res Ther.

[3] Nzeusseu Toukap A, Galant C, Theate I, Maudoux AL, Lories RJ, Houssiau FA (2007). Identification of distinct gene expression profiles in the synovium of patients with systemic lupus erythematosus. Arthritis Rheum.

[4] Lauwerys BR, Hernández-Lobato D, Gramme P, Ducreux J, Dessy A, Focant I (2015). Heterogeneity of synovial molecular patterns in patients with arthritis. PLoS One.

[5] Gutierrez-Roelens I, Galant C, Theate I, Lories RJ, Durez P, Nzeusseu-Toukap A (2011). Rituximab treatment induces the expression of genes involved in healing processes in the rheumatoid arthritis synovium. Arthritis Rheum.

[6] Ducreux J, Durez P, Galant C, Nzeusseu Toukap A, Van den Eynde B, Houssiau FA (2014). Global molecular effects of tocilizumab therapy in rheumatoid arthritis synovium. Arthritis Rheumatol.

[7] Badot V, Galant C, Nzeusseu Toukap A, Theate I, Maudoux AL, Van den Eynde BJ (2009). Gene expression profiling in the synovium identifies a predictive signature of absence of response to adalimumab therapy in rheumatoid arthritis. Arthritis Res Ther.

[8] Badot V, Durez P, Van den Eynde BJ, Nzeusseu-Toukap A, Houssiau FA, Lauwerys BR (2011). Rheumatoid arthritis synovial fibroblasts produce a soluble form of the interleukin-7 receptor in response to pro-inflammatory cytokines. J Cell Mol Med.

[9] Huang DW, Sherman BT, Lempicki RA (2009). Systematic and integrative analysis of large gene lists using DAVID bioinformatics resources. Nat Protoc.

[10] Smiljanovic B, Grün JR, Biesen R, Schulte-Wrede U, Baumgrass R, Stuhlmüller B (2012). The multifaceted balance of TNF-α and type I/II interferon responses in SLE and RA: how monocytes manage the impact of cytokines. J Mol Med (Berl).

[11] Cornish T, Morgan J, Gurel B, De Marzo AM (2008). FrIDA: An open source framework for image dataset analysis. Arch Pathol Lab Med.

[12] Haringman JJ, Vinkenoog M, Gerlag DM, Smeets TJ, Zwinderman AH, Tak PP (2005). Reliability of computerized image analysis for the evaluation of serial synovial biopsies in randomized controlled trials in rheumatoid arthritis. Arthritis Res Ther.

[13] Du F, Wang L, Zhang Y, Jiang W, Sheng H, Cao Q (2008). Role of GADD45 beta in the regulation of synovial fluid T cell apoptosis in rheumatoid arthritis. Clin Immunol.

[14] Svensson CI, Inoue T, Hammaker D, Fukushima A, Papa S, Franzoso G (2009). Gadd45beta deficiency in rheumatoid arthritis: enhanced synovitis through JNK signaling. Arthritis Rheum.

[15] Kavanaugh A, Mease PJ, Gomez-Reino JJ, Adebajo AO, Wollenhaupt J, Gladman DD (2014). Treatment of psoriatic arthritis in a phase 3 randomised, placebo-controlled trial with apremilast, an oral phosphodiesterase 4 inhibitor. Ann Rheum Dis.

[16] Genovese MC, Jarosova K, Cieślak D, Alper J, Kivitz A, Hough DR (2015). Apremilast in Patients With Active Rheumatoid Arthritis: A Phase II, Multicenter, Randomized, Double-Blind, Placebo-Controlled. Parallel-Group Study. Arthritis Rheumatol.

[17] Kelly S, Humby F, Filer A, Ng N, Di Cicco M, Hands RE (2015). Ultrasound-guided synovial biopsy: a safe, well-tolerated and reliable technique for obtaining high-quality synovial tissue from both large and small joints in early arthritis patients. Ann Rheum Dis.

[18] Humby F, Kelly S, Bugatti S, Manzo A, Filer A, Mahto A (2016). Evaluation of Minimally Invasive, Ultrasound-guided Synovial Biopsy Techniques by the OMERACT Filter-determining Validation Requirements. J Rheumatol.

