# Supplementary material for: Higher expression of TNFα-induced genes in the synovium of patients with early rheumatoid arthritis correlates with disease activity, and predicts absence of response to first line therapy
Source: Arthritis Res Ther. 2016 Jan 20;18:19. doi: 10.1186/s13075-016-0919-z (PMC4719339; doi:10.1186/s13075-016-0919-z)
Supplement: Additional file 1: — Patients’ characteristics (immunohistochemistry studies on needle arthroscopic biopsies). Characteristics of the patients with early rheumatoid arthritis (RA) included in the immunohistochemistry studies on needle arthroscopic biopsies. (DOCX 56 kb) [file 13075_2016_919_MOESM1_ESM.docx]

**Additional file 1: Patients’ characteristics (immunohistochemistry studies on needle arthroscopic biopsies)**

| **Early untreated RA patients (n=46)** | |
| --- | --- |
| Age at baseline (mean ± SD years) | 49.1 ± 14.7 |
| Gender (females/males) | 38/8 |
| ACPA status (% positive) | 73.9% |
| RF status (% positive) | 78.2% |
| Disease duration (mean ± SD months) | 6.4 ± 4.4 |
| **Treatment initiated after baseline biopsy** |  |
| Methotrexate monotherapy (15-20 mg/week) | 17 |
| Original biological DMARDs (Infliximab, Tocilizumab) monotherapy | 21 |
| Methotrexate (15-20 mg/week) + original biological DMARD (Adalimumab, Certolizumab pegol, Golimumab, CTLA4-Ig) combination therapy | 8 |
| **DAS28CRP** |  |
| Baseline | 4.68 ± 1.12 |
| After 3 months | 3.07 ± 1.41 |
| After 6 months | 2.90 ± 1.47 |
| EULAR Good-, moderate-, poor-responders (n, n, n) at 6 months | 22, 9, 15 |
