# Supplementary material for: Higher expression of TNFα-induced genes in the synovium of patients with early rheumatoid arthritis correlates with disease activity, and predicts absence of response to first line therapy
Source: Arthritis Res Ther. 2016 Jan 20;18:19. doi: 10.1186/s13075-016-0919-z (PMC4719339; doi:10.1186/s13075-016-0919-z)
Supplement: Additional file 2: — Patients’ characteristics (immunohistochemistry studies on ultrasound (US)-guided biopsies). Characteristics of the patients with early rheumatoid arthritis (RA) included in the immunohistochemistry studies on US-guided biopsies. (DOCX 47 kb) [file 13075_2016_919_MOESM2_ESM.docx]

**Additional file 2: Patients’ characteristics (immunohistochemistry studies on US-guided biopsies)**

| **Early untreated RA patients (n=35)** | |
| --- | --- |
| Age at baseline (mean ± SD years) | 54.3 ± 17.4 |
| Gender (females/males) | 24/11 |
| ACPA status (% positive) | 71.4% |
| RF status (% positive) | 71.4% |
| Disease duration (mean ± SD months) | 5.5 ± 3.4 |
| **Treatment initiated after baseline biopsy** |  |
| Methotrexate monotherapy (15-20 mg/week) | 35 |
| **DAS28CRP** |  |
| Baseline | 6.37 ± 1.12 |
| After 3 months | 3.98 ± 2.38 |
| After 6 months | 3.67 ± 2.23 |
| EULAR responders, non-responders (n, n) at 6 months | 20, 15 |
